# Hemodynamic characterization of cardiac dysfunction after traumatic brain injury using a controlled cortical impact model in male rats

**DOI:** 10.14814/phy2.71055

**Published:** 2026-08-16

**Authors:** Preston So, Amit Iyengar, Noah Weingarten, Mrinal Patel, Jessica Dominic, Andrew Belec, Joyce Ho, Samuel S. Shin, Pavan Atluri

**Affiliations:** ^1^ Drexel University College of Medicine Philadelphia Pennsylvania USA; ^2^ Department of Cardiovascular Surgery University of Pennsylvania Perelman School of Medicine Philadelphia Pennsylvania USA; ^3^ Department of Cardiac Surgery Thomas Jefferson University Philadelphia Pennsylvania USA; ^4^ Department of Neurocritical Care University of Pennsylvania Perelman School of Medicine Philadelphia Pennsylvania USA

**Keywords:** cardiac dysfunction, cortical impact, heart failure, traumatic brain injury

## Abstract

Traumatic brain injury (TBI) is associated with cardiovascular dysfunction that worsens clinical outcomes, yet the underlying mechanisms are poorly understood. Clinical studies are limited by heterogeneity, highlighting the need for controlled experimental models. Controlled cortical impact (CCI) provides precise control of injury severity, and invasive pressure‐volume (PV) loop analysis allows detailed hemodynamic assessment. Adult male Wistar rats underwent sham operation or mild or severe CCI‐induced TBI. PV‐loop assessment and transthoracic echocardiography were performed at 8 or 24 h after TBI or sham. Serum epinephrine (EPI) and norepinephrine (NE) were quantified by ELISA. At 8 h post‐injury, mild TBI was associated with decreased E_ES_ (*p* = 0.013) and increased V_120_ (*p* = 0.016), accompanied by qualitative global hypokinesis on echocardiography. Severe TBI demonstrated decreased cardiac output (*p* = 0.009) and stroke work (*p* = 0.006), whereas load‐independent systolic indices were unchanged. At 24 h post‐injury, severe TBI exhibited persistent global cardiac dysfunction. Serum NE and EPI were similar to shams; however, the NE‐to‐EPI ratio was decreased 8 h after mild TBI (*p* = 0.042). These findings suggest that TBI is associated with cardiovascular dysfunction that varies with injury severity; however, temporal characterization following mild TBI requires further study. Controlled experimental models provide a valuable platform for elucidating brain‐heart interactions after TBI.

## 1 BACKGROUND

Traumatic brain injury (TBI) imposes a substantial public health burden worldwide, with an annual global incidence exceeding 20 million, and United States costs estimated at ~40.6 billion US dollars annually (Miller et al., 2021). The severity of injury, often graded from mild to severe TBI, is an important driver of healthcare utilization and clinical trajectory. An estimated 70%–90% of TBIs presenting to the emergency department are mild (concussions); however, many cases of mild TBI never seek medical care (Cassidy et al., 2004; Daugherty et al., 2024). In contrast, moderate and severe TBI patients often require prolonged hospitalization and experience high rates of complications, functional impairment, and mortality (Al‐Hassani et al., 2018; Fuller et al., 2016).

Extracranial organ dysfunction following TBI may substantially worsen clinical outcomes, particularly when the cardiovascular system is affected. TBI‐associated cardiac dysfunction impairs systemic and cerebral perfusion, potentially exacerbating secondary brain injury and extracranial organ ischemia (Krishnamoorthy et al., 2017; Krishnamoorthy et al., 2021; Yan et al., 2025). Although TBI severity has been associated with the development of cardiac dysfunction, the reported incidence is highly variable. Prospective studies have demonstrated rates of abnormal echocardiographic findings (including regional wall motion abnormalities and reduced ejection fraction) between 13% and 42% (Hasanin et al., 2016; Krishnamoorthy et al., 2017; Venkata & Kasal, 2018). Additionally, one prospective study found that cardiac dysfunction following severe TBI was independently associated with in‐hospital mortality (Hasanin et al., 2016).

Cardiac dysfunction develops within a broad multisystem response to brain injury. The primary mechanical insult triggers an exaggerated sympathetic and inflammatory state, leading to surges in catecholamines (epinephrine and norepinephrine), glucocorticoids, and proinflammatory cytokines (Kinoshita, 2016). At the same time, compensatory mechanisms such as cerebral autoregulation and the Cushing reflex act to preserve cerebral perfusion in the setting of rising intracranial pressure (ICP) (Coppalini et al., 2024; Molina, 2005). Amidst this physiologic process, cardiac dysfunction may occur through mechanisms that remain incompletely understood.

Despite increasing recognition, the clinical phenotypes of TBI‐associated cardiac dysfunction are ill‐defined. Clinical studies have described TBI‐associated cardiac dysfunction with nonspecific features, including ECG changes, echocardiographic abnormalities, and hemodynamic instability (Ediga et al., 2024; Venkata & Kasal, 2018). Further, inherent heterogeneity in clinical settings, where variables such as timing, injury severity, and interventions are challenging to control, has led to conflicting results across studies (Chaikittisilpa et al., 2024; Hilz et al., 2011; Krishnamoorthy et al., 2022; Lenstra et al., 2021; Serri et al., 2016; Siegel et al., 2024) and limits the ability to isolate causal mechanisms. Therefore, controlled experimental models are essential for advancing our understanding of TBI‐associated cardiac dysfunction.

Rodent studies provide a controlled platform for the hemodynamic investigation of both TBI and cardiac disease, with well‐validated applications across both domains (Balakin et al., 2024; Dixon et al., 1991; Gomes et al., 2013). The controlled cortical impact (CCI) model is well established in rodent TBI research (Dixon et al., 1991; Fournier et al., 2021; Jamnia et al., 2017), allowing for precise, reproducible induction of TBI through quantitative control of injury parameters (velocity, depth, and dwell time). In parallel, invasive pressure‐volume (PV) loop analysis provides continuous, in vivo hemodynamic monitoring with measurement of both load‐dependent and load‐independent indices of cardiac function (Miranda‐Silva et al., 2021; Protti et al., 2024). Together, these approaches are well‐suited to characterize severity‐dependent effects of TBI under controlled experimental conditions.

## 2 MATERIALS AND METHODS

### 2.1 Animals and ethical approval

All experiments were approved by the Institutional Animal Care and Use Committee (IACUC) at the University of Pennsylvania (Protocol no. 806689) and conducted in accordance with the National Institutes of Health Guide for Care and Use of Laboratory Animals (National Research Council (US) Committee for the Update of the Guide for the Care and Use of Laboratory Animals, 2011). Adult male Wistar rats (8–10 weeks, 300–500 g; Charles River Laboratories, Boston, MA, USA) were acclimated to in‐house facilities with access to ad libitum food (Laboratory Rodent Diet 5001, catalog no. 0001319, LabDiet, St. Louis, MO, USA) and water for at least 1 week prior to experiments.

### 2.2 Study design

**FIGURE 1:**
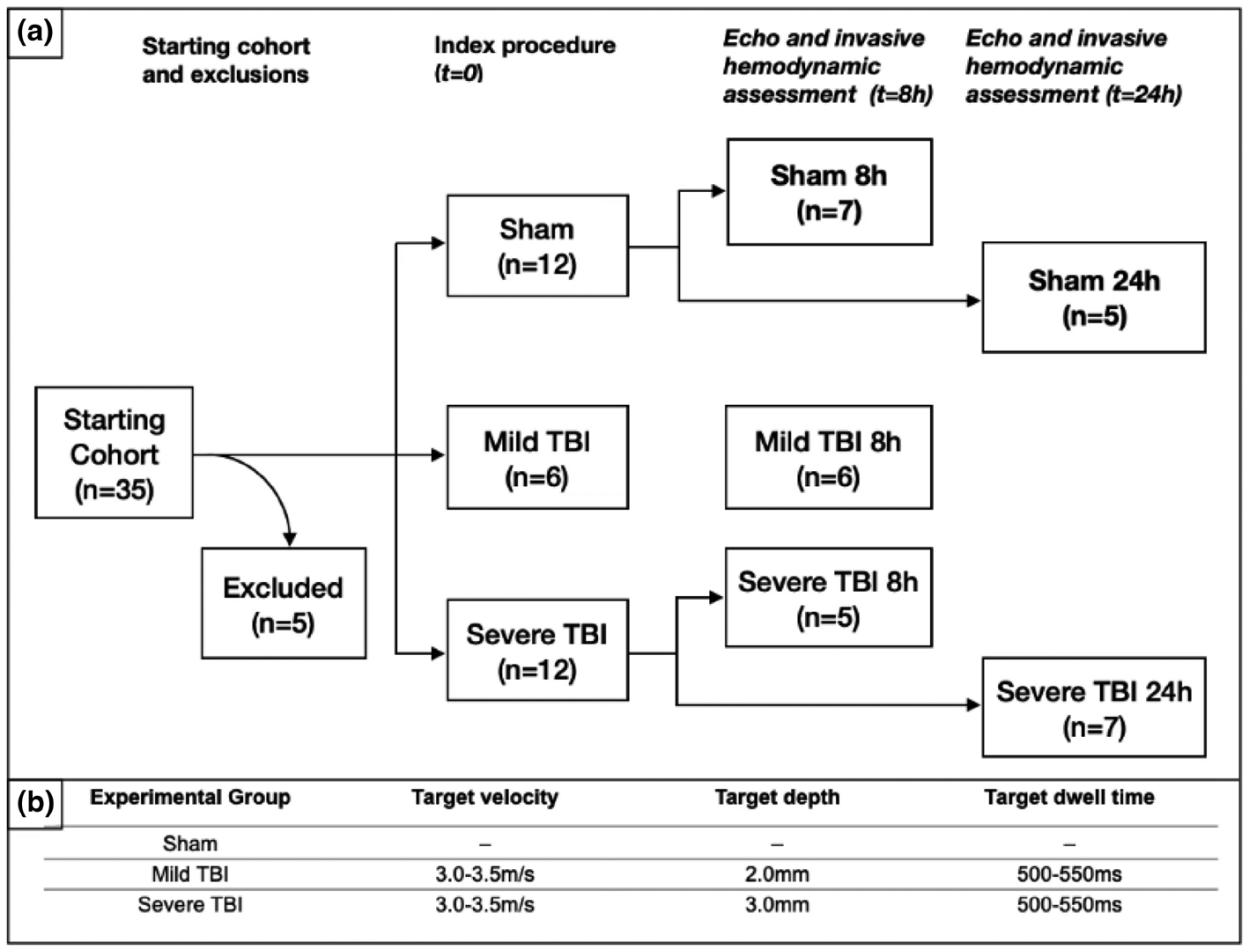
Rodent fixation and positioning for controlled cortical impact.

Rats (Figure 1a) were randomly assigned to five groups (*n* = 7/group) stratified by TBI and severity (sham, mild TBI, severe TBI) and timing of hemodynamic analysis (8 or 24 h). The 8 h cohorts included sham, mild TBI, and severe TBI groups. The 24 h cohorts included sham and severe TBI groups. Animals underwent the index procedure (sham or TBI) and then returned for echocardiography and invasive hemodynamic (PV loop) analysis at the specified timepoint. Blood was then collected for quantification of catecholamines. Exclusions were made prior to data analysis, based on predefined criteria and recommendations in the literature (Wearing et al., 2025). Animals were excluded for mortality (e.g., prior to PV loop assessment), profound hemodynamic instability (e.g., prohibiting preload‐reduction), or technical artifact during PV acquisition (e.g., non‐physiologic PV loop waveform).

### 2.3 Sham operation

Animals were weighed and then anesthetized with isoflurane (4% induction, 2% maintenance in 100% oxygen), orotracheally intubated with a 16G angiocatheter and connected to a rodent mechanical ventilator (Hallowell EMC, Pittsfield, MA). Rodents were kept on a heating pad to maintain core body temperature, subcutaneous meloxicam (4 mg/kg) was administered, and sedation was confirmed by toe pinch. After shaving and sterilizing the dorsal head, a 3 cm sagittal scalp incision was made to expose the cranium and identify right parietal suture. Finally, bupivacaine (0.15 mg/kg, subcutaneous) was given, incisions were reapproximated by primary intention, anesthesia was weaned, and animals recovered with standard monitoring. 8 h cohorts were fasted prior to hemodynamic analysis, and 24 h cohorts were given ad libitum food and water to avoid fasting‐associated hemoconcentration (Kale et al., 2009).

### 2.4 TBI using the controlled‐cortical impact device (CCI)

Animals in the TBI groups were prepped and the right parietal suture was identified as described previously. Next, a craniectomy was made between lambda and bregma using a bone drill to expose the right cerebral cortex, and the head was secured to the CCI apparatus, with exposed brain positioned directly beneath the impact tip. TBI force parameters (Figure 1b) were selected within ranges previously documented in rat TBI models (Adelson et al., 2013; Osier & Dixon, 2016; Washington et al., 2012). After calibration and positioning checks, the impact was delivered and the incision was closed. Postoperative analgesia, recovery, and fasting conditions were maintained as described above.

### 2.5 Transthoracic echocardiography (TTE)

At the specified 8 or 24 h timepoint, animals were re‐anesthetized, intubated, and ventilated as detailed previously. After shaving the chest, TTE was performed using a 12‐MHz transducer with built‐in analysis. Short‐axis images were used to obtain LV end‐diastolic and end‐systolic diameters (LVEDD and LVESD) and to estimate ejection fraction (EF) and stroke volume (SV). Parasternal long‐axis images were used to assess LV motion qualitatively and measure left ventricular outflow tract diameter (LVOTd). Wall motion abnormalities were categorized descriptively (e.g., global hypokinesis vs. apical hypokinesis).

### 2.6 Invasive hemodynamic assessment via pressure‐volume analysis

A 1.9F conductance PV catheter (Transonic Systems, Ithaca, NY) was calibrated by two‐point calibration with TTE‐derived LV volumes. Data were recorded at 1 kHz using PowerLab and LabChart Pro data acquisition software (ADInstruments, Colorado Springs, CO) and analyzed with LabChart Pro (Ogilvie et al., 2020).

After TTE, the right common carotid artery was exposed via right lateral neck dissection, an arteriotomy was made, and the catheter was advanced retrograde into the left ventricle. To reduce artifact associated with positive‐pressure ventilation, pleural and mediastinal cavities were exposed bilaterally, and ventilation was paused immediately prior to hemodynamic measurements. For inferior vena cava (IVC) occlusions, the IVC was exposed through a midline laparotomy.

Three separate baseline measurements were recorded for at least 10 cardiac cycles under apnea, separated by at least 5 min of equilibration. Baseline measurements were recorded in triplicate and averaged for each animal for assessment of global and load‐dependent cardiac function. IVC occlusions were performed by direct compression immediately caudal to the liver using a mosquito hemostat. Three separate occlusions were performed per animal, each separated by at least 10 min of equilibration, and averaged.

### 2.7 Sample collection and quantification of serum catecholamines

Arterial blood (~3 mL) was collected into serum separator tubes and allowed to coagulate for 10 min, centrifuged at 2900*g* for 10 min, and serum was stored at −80°C. Serum epinephrine (EPI) and norepinephrine (NE) were quantified by enzyme‐linked immunosorbent assay (ELISA) according to the manufacturer's instructions (3‐CAT ELISA Fast Track, catalog no. BA E‐6600R, LDN Immunoassays and Services, Nordhorn, Germany). Animals were euthanized by bilateral thoracotomy and cardiectomy after sample collection.

### 2.8 Data processing

Pressure and volume signals were processed into hemodynamic indices in real time. Baseline volume data and IVC occlusion data were further processed post hoc to minimize inter‐animal variability. Volume indices obtained from baseline recordings were indexed to body surface area using Meeh's formula ${\left(9.83\times \text{rodent mass}\right)}^{2/3}$ (Gouma et al., 2012). IVC occlusion data were restricted to segments with physiologic waveform achieving a 30%–40% reduction in V_ed_ relative to baseline. Further, segments where P_es_ dropped below 40 mmHg were excluded to minimize the effects of reflex sympathetic activation and coronary ischemia (Wearing et al., 2025).

### 2.9 Data analysis

Cardiovascular function was evaluated with respect to global cardiac performance, systolic and diastolic function, and ventriculoarterial coupling parameters recorded on invasive hemodynamic assessment. PV loops were interpreted by two independent researchers. Global cardiac performance indices included cardiac output (CO), stroke work (SW), stroke volume (SV), and heart rate (HR). Load‐dependent systolic indices included ejection fraction (EF), end‐systolic pressure and volume (P_es_, V_es_) and maximum rates of LV pressure increase (dP/dt_max_) and volume decrease (dV/dt_min_). Load‐dependent diastolic indices included end‐diastolic pressure and volume (P_ed_, V_ed_), maximal rates of LV volume increase (dV/dt_max_) and pressure decrease (dP/dt_min_) and the time constant of relaxation (τ, Glantz method).

Load‐independent systolic indices included end‐systolic elastance (E_ES_), defined as the slope of the linear portion of the end‐systolic pressure‐volume relationship (ESPVR [P_es_ = E_ES_ · (V_es_ – V_0_)]), and the ESPVR‐derived end‐systolic volume at 120 mmHg (V_120_). Load‐independent diastolic indices included the diastolic stiffness coefficient (β) measured from an exponential fit (P_ed_ = α·e^β ·Ved^) of the end‐diastolic pressure volume relationship (EDPVR), and the EDPVR‐derived end‐diastolic volume at 30 mmHg (V_30_). Ventriculoarterial coupling was assessed using arterial elastance (E_A_) and the ventriculoarterial coupling coefficient (E_A_/E_ES_).

### 2.10 Statistical analysis

The primary comparison evaluated 8 h mild and severe TBI versus sham cohorts at 8 h. Secondary analysis compared severe TBI vs. controls at 8 and 24 h. The primary comparison was analyzed using one‐way ANOVA with Dunnett's post hoc correction. The secondary model was analyzed using two‐way ANOVA with Sidak's multiple comparisons test.

Data were evaluated for normality using the Shapiro–Wilk test and heteroscedasticity using the Brown‐Forsyth test. When heteroscedasticity was significant, Welch's ANOVA with Dunnett's T3 post hoc correction was applied. Non‐parametric data were analyzed using Kruskal–Wallis with Dunn's post‐hoc correction. E_ES_ was evaluated using multiple linear regression in both the 8 h and 24 h cohorts, incorporating group, volume‐axis intercept (V_0_), and group × V_0_ interaction terms. Findings were corroborated with V_120_ comparison as previously described (Burkhoff et al., 2005).

Descriptive statistics are expressed as mean ± standard deviation or median [IQR]. Statistical significance was set at *p* < 0.05. Statistical analyses and data visualization were performed using GraphPad Version 10.3.0 (GraphPad Software Inc., Boston, MA).

## 3 RESULTS

### 3.1 Cohort characteristics

Of the 35 animals instrumented, 30 were included in the final analysis. Five rodents were excluded due to intraoperative mortality (*n* = 1), anesthesia intolerance (*n* = 2), or technical artifact during invasive hemodynamic analysis (*n* = 2). Rodent weights were similar across groups (all *p* > 0.05).

### 3.2 PV‐loop assessment: 8 h after mild and severe TBI

**FIGURE 2:**
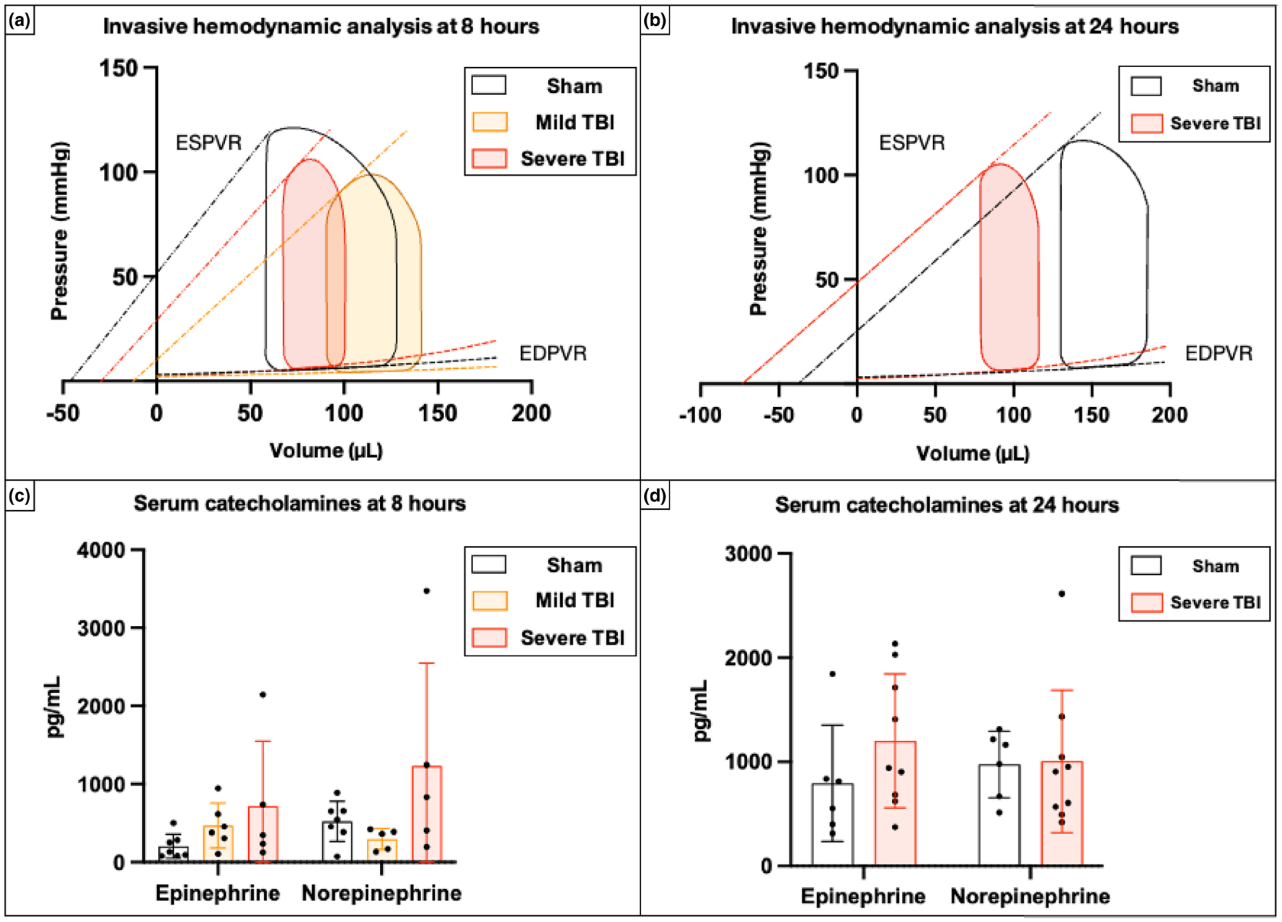
Invasive hemodynamic assessment and serum catecholamines 8 h and 24 h after TBI. Cohort‐averaged pressure‐volume loops obtained by left ventricular catheterization in sham, mild, and severe TBI cohorts at 8 h (a) and 24 h (b). Loops represent cohort mean values during steady‐state recordings, with volumes fixed at each cohort's end‐systolic and ‐diastolic volumes. End‐systolic and end‐diastolic pressure‐volume relationships (ESPVR and EDPVR) were derived from inferior vena cava (IVC) occlusions. ESPVR was modeled using a linear relationship (P_es_ = E_ES_ · [V_es_ – V_0_]) and EDPVR was fitted using an exponential model (P_ed_ = α·e^β ·Ved^). Serum catecholamine concentrations were measured at 8 h (c) and 24 h (d) after injury. Norepinephrine and epinephrine were quantified by direct ELISA.

Hemodynamic data and cohort‐averaged PV‐loops for the 8 h cohorts are shown in Table 1 and Figure 2a, respectively. At 8 h after injury, mild TBI demonstrated a 20% decrease in CO (*p* = 0.058) and a 19% decrease in SW (*p* = 0.051), although neither reached significance. SV and HR were similar between mild TBI and sham groups. Load‐dependent systolic indices demonstrated reduced EF (*p* = 0.026), and load‐independent contractility indices showed decreased E_ES_ (*p =* 0.013) and increased V_120_ (*p* = 0.016). V_ED_ and E_A_ were unchanged. Diastolic function indices, including relaxation (dP/dt_min_, τ) and load‐independent compliance indices (ß, V_30_) did not differ from shams. Ventriculoarterial coupling was unaffected.

**TABLE 1:** 8 h Invasive hemodynamic analysis, echocardiography, and serum catecholamines.

Variable	Sham (*n* = 7)	Mild TBI (*n* = 6)		Severe TBI (*n* = 5)	
	Mean ± SD	Mean ± SD	*p* Value	Mean ± SD	*p* Value
Animal mass, g, median (IQR)	341 (316–406)	376 (371–436)	0.16	330 (325–350)	0.87
Global cardiac performance indices					
CO, μL/min·cm^2^	52 ± 7	41 ± 11	0.058	36 ± 4	0.009
SW, mmHg·μL/cm^2^, median (IQR)	15 (14–19)	12 (10–13)	0.052	9.9 (8.7–12)	0.006
SV, μL/cm^2^	0.15 ± 0.03	0.13 ± 0.039	0.30	0.1 ± 0.018	0.029
HR, beats per minute	341 ± 31	325 ± 49	0.75	355 ± 48	0.81
Systolic indices					
EF, %, median (IQR)	57 (53–59)	44 (42–48)	0.026	46 (37–47)	0.087
P_es_, mmHg	116 ± 15	100 ± 9	0.047	103 ± 12	0.14
V_es_, μL/cm^2^	0.13 ± 0.04	0.17 ± 0.047	0.13	0.14 ± 0.05	0.69
dP/dt_max_, mmHg/s	6567 ± 1355	5296 ± 1634	0.29	5195 ± 390	0.073
dV/dt_min_, –μL/s·cm^2^	6.2 (5.2–6.6)	4.9 (4.2–5.4)	0.52	3.7 (3.3–3.8)	0.012
E_ES_, mmHg/μL	1.76 ± 1.44	1.01 ± 0.52	0.013	1.08 ± 0.22	0.15
V_120_, μL	61 ± 22	133 ± 62	0.016	92 ± 38	0.39
V_0_, μL	−46 ± 41	−12 ± 37	0.17	−30 ± 12	0.64
Diastolic indices					
P_ed_, mmHg	6.9 ± 1.3	7.4 ± 2.6	0.90	7.6 ± 3.1	0.80
V_ed_, μL/cm^2^	0.26 ± 0.05	0.26 ± 0.05	0.98	0.21 ± 0.07	0.27
dPdt_min_, –mmHg/s, median (IQR)	7171 (6245–8300)	6056 (4790–6816)	0.16	6482 (5719–7038)	0.74
dV/dt_max_, μL/s·cm^2^	6.4 ± 1.6	6.0 ± 1.8	0.85	4.4 ± 0.59	0.071
τ, ms	12.2 ± 1.8	13.4 ± 3	0.57	12.1 ± 1.9	>0.99
α	3.3 ± 1.5	2.5 ± 2	0.68	3.1 ± 2.5	0.97
ß	0.007 ± 0.005	0.011 ± 0.012	>0.99	0.017 ± 0.018	0.32
V_30_	405 ± 201	484 ± 332	>0.99	244 ± 89	0.20
Arterial elastance and ventriculoarterial coupling					
E_A_, mmHg/μL	1.79 ± 0.56	1.75 ± 0.82	0.99	2.38 ± 0.33	0.21
E_A_/E_ES_	1.41 ± 0.69	1.99 ± 1.07	0.38	2.3 ± 0.65	0.15
Echocardiography					
LVESD, cm, median (IQR)	0.39 (0.35–0.50)	0.42 (0.41–0.50)	0.83	0.48 (0.44–0.49)	0.99
LVEDD, cm	0.71 ± 0.13	0.7 ± 0.07	0.98	0.74 ± 0.06	0.90
FS, %	40.4 ± 7	36.3 ± 8.2	0.61	39.8 ± 9.5	>0.99
LVOTd, cm	0.27 ± 0.01	0.27 ± 0.02	0.99	0.28 ± 0.01	0.43
V_es_, μL, median (IQR)	22.0 (17.0–36.5)	55.0 (37.3–61.5)	0.14	29.0 (23.0–33.3)	0.99
V_ed_, μL	107.1 ± 38.5	123.5 ± 10.9	0.44	77.3 ± 9.8	0.13
SV, μL	76.7 ± 21.9	67.5 ± 42.4	0.99	50 ± 7.9	0.071
EF, %	73.3 ± 8	60.4 ± 14.7	0.098	65.2 ± 11.2	0.42
Mild global HK, *n* (%)	0 (0)	3 (50)		1 (16.7)	
Apical HK, *n* (%)	0 (0)	0 (0)		1 (16.7)	
Normal LV wall motion, *n* (%)	7 (100)	3 (50)		4 (66.7)	
Serum catecholamines					
Total (NE + EPI), pg/mL	725 ± 370	764 ± 346	>0.99	1948 ± 2146	0.18
NE, pg/mL	522 ± 257	295 ± 134	0.82	1230 ± 1318	0.21
EPI, pg/mL	203 ± 155	467 ± 288	0.52	718 ± 830	0.14
NE:EPI	3.01 (1.98–5.78)	0.41 (0.35–1.38)	0.042	1.68 (1.62–1.72)	0.46

*Note*: Values are presented as mean ± standard deviation unless otherwise specified. Volume‐derived measurements are indexed to body surface area. Steady state hemodynamics were assessed using one‐way ANOVA with Dunnett's post‐hoc correction for parametric data, or Kruskal–Wallis testing with Dunn's post‐hoc correction for nonparametric data, comparing Mild TBI and Severe TBI groups. EES was compared using a multiple linear regression model incorporating interaction terms for TBI severity and V_0_.

Severe TBI at 8 h demonstrated a 30% decrease in CO (*p* = 0.009), a 34% decrease in SW (*p* = 0.006), and a 33% decrease in SV (*p* = 0.030). dV/dt_min_ was decreased (*p =* 0.012). EF, dP/dt_max_, and dV/dt_max_ were lower, but did not reach significance (*p* = 0.087, *p* = 0.073, and *p* = 0.071, respectively). Load‐independent systolic indices (E_ES_ and V_120_), dP/dt_min_ and τ were similar.

Load‐independent compliance indices demonstrated a 57% increase in β, and a 32% decrease in V_30_, although neither difference reached significance (*p =* 0.318 and *p* = 0.196, respectively).

### 3.3 PV‐loop assessment: 24 h after severe TBI

Hemodynamic data and cohort‐averaged PV‐loops for the 24 h cohorts are displayed in Table 2 and Figure 2b, respectively. At 24 h, severe TBI demonstrated a similar reduction in global cardiac performance that was observed at 8 h. CO and SW were decreased (CO: *p* = 0.020; SW: *p* = 0.029), V_ES_ and V_ED_ decreased (*p* = 0.005 and *p* = 0.003, respectively), whereas SV and systolic function indices were similar. dV/dt_max_ was reduced (*p* = 0.043), while dP/dt_min_, τ, and load‐independent diastolic indices were unchanged.

**TABLE 2:** 24 h Invasive hemodynamic analysis, echocardiography and serum catecholamines.

Variable	Sham 24 h (*n* = 5)	Severe TBI 24 h (*n* = 7)	
	Mean ± SD	Mean ± SD	*p* Value
Animal mass, g	383 ± 51	381 ± 32	>0.99
Global cardiac performance indices			
CO, μL/min·cm^2^	53 ± 3.2	40 ± 12	0.020
SW, mmHg·μL/cm^2^	16.5 ± 2.5	11.2 ± 2.8	0.029
SV, μL/cm^2^	0.15 ± 0.02	0.12 ± 0.03	0.11
HR, beats per minute	358 ± 47	339 ± 53	0.72
Systolic indices			
EF, %	42 ± 3.9	46 ± 6.8	0.60
P_es_, mmHg	113 ± 14	101 ± 15	0.28
V_es_, μL/cm^2^	0.25 ± 0.04	0.16 ± 0.06	0.005
dP/dt_max_, mmHg/s	5586 ± 1067	5521 ± 828	0.99
dV/dt_min_, –μL/s·cm^2^	5.6 ± 1.7	4.1 ± 1.7	0.14
E_ES_, mmHg/μL	1.1 ± 0.5	1.5 ± 1.4	0.62
V_120_, μL	114 ± 62	100 ± 67	0.87
V_0_, μL	−38 ± 48	−74 ± 89	0.52
Diastolic indices			
P_ed_, mmHg, median (IQR)	8.4 (6.7–8.4)	6.3 (4.8–6.4)	0.55
V_ed_, μL/cm^2^	0.36 ± 0.06	0.23 ± 0.06	0.003
dPdt_min_, –mmHg/s	7429 ± 1872	6717 ± 1610	0.80
dV/dt_max_, μL/s·cm^2^	6.8 ± 1.6	4.4 ± 1.9	0.032
τ, ms	12.1 ± 2.8	13.2 ± 1.6	0.60
α	3.2 ± 1.5	2.5 ± 0.9	0.73
ß	0.007 ± 0.005	0.010 ± 0.006	0.80
V_30_	489 ± 228	355 ± 233	0.51
Arterial elastance and ventriculoarterial coupling			
E_A_, mmHg/μL	1.58 ± 0.4	1.71 ± 0.77	0.92
E_A_/E_ES_	1.77 ± 0.83	1.95 ± 1.19	0.94
Echocardiography			
LVESD, cm	0.48 ± 0.1	0.4 ± 0.04	0.065
LVEDD, cm	0.75 (0.72–0.77)	0.64 (0.60–0.66)	0.20
FS, %	33.1 ± 6.1	37.9 ± 3.8	0.13
LVOTd, cm	0.30 ± 0.02	0.29 ± 0.02	0.99
V_es_, μL	41.8 ± 10.3	31.9 ± 15.8	0.23
V_ed_, μL	118.4 ± 17.3	97.4 ± 28.4	0.15
SV, μL	73.5 ± 9.3	67.5 ± 17	0.51
EF, %	64.9 ± 5.8	68.6 ± 9.5	0.46
Mild global HK, *n* (%)	0 (0)	1 (14.3)	
Apical HK, *n* (%)	1 (20)	1 (14.3)	
Normal LV wall motion, *n* (%)	4 (80)	5 (71.4)	
Serum catecholamines			
Total (NE + EPI), pg/mL	1828 ± 672	2277 ± 1342	0.81
NE, pg/mL	938 ± 343	1076 ± 768	>0.99
EPI, pg/mL	890 ± 564	1201 ± 679	0.36
NE:EPI	0.93 (0.8–1.62)	0.6 (0.56–1.12)	0.92

*Note*: Values are presented as mean ± standard deviation unless otherwise specified. All volume‐derived measurements are indexed to body surface area. Group differences were assessed using two‐way ANOVA with Šidák‐adjusted pairwise comparisons. EES was compared using a multiple linear regression model incorporating interaction terms for group (sham vs. severe TBI) and V_0_.

### 3.4 Echocardiography

There were no significant differences in LVEDD, LVESD, fractional shortening, or LVOTd between shams and mild or severe TBI at 8 h. V_ES_, V_ED_, EF, and SV demonstrated similar trends to those observed with invasive hemodynamic assessment, although none reached statistical significance. At 24 h, LVESD tended to decrease in severe TBI (*p =* 0.065) whereas other TTE parameters were unchanged.

Qualitative assessment of LV motion abnormalities in the 8 h groups demonstrated mild global hypokinesis in 50% (*n* = 3) of the mild TBI group. In the severe TBI group, one rodent demonstrated mild global hypokinesis and one showed apical hypokinesis at both 8 and 24 h. No wall motion abnormalities were observed in the sham group at 8 h; however one sham rodent had apical hypokinesis at 24 h.

### 3.5 Serum catecholamines

Serum EPI, NE (Figure 2c,d), and total catecholamine concentrations (Figure 6) were not significantly different than shams in TBI groups at 8 and 24 h. Interestingly, the NE‐to‐EPI ratio (NE:EPI) decreased by 86% after mild TBI (*p* = 0.042) but did not differ in severe TBI at 8 or 24 h.

## 4 DISCUSSION

TBI disrupts the brain–heart axis, and the phenotype of cardiac dysfunction likely depends on injury severity and time after injury; however, controlling these variables in clinical studies is challenging. Although prior animal models have advanced our understanding, important knowledge gaps remain. In this study, we used a CCI model to examine cardiovascular function at 8 h and 24 h using PV loop analysis. We observed hemodynamic alterations following severe TBI at both time points and identified changes in load‐independent systolic indices following mild TBI. Despite these functional changes, circulating NE and EPI levels were similar across groups, suggesting that circulating catecholamines alone do not fully explain the observed cardiovascular derangements.

### 4.1 Systolic dysfunction

Acute systolic dysfunction has been reported as an extracranial complication of TBI, but evidence linking TBI to systolic dysfunction is inconsistent. Clinical studies present conflicting findings, including reports that TBI severity correlates with systolic dysfunction (Krishnamoorthy et al., 2017; Mazandi et al., 2025; Venkata & Kasal, 2018), and others that found no clear relationship (Cuisinier et al., 2016; Serri et al., 2016). Additionally, mild TBI is rarely associated with overt systolic dysfunction but has been described with autonomic dysfunction and electrocardiographic abnormalities (Ediga et al., 2024; Esterov & Greenwald, 2017; Siegel et al., 2024), making it unclear whether LV dysfunction occurs with mild injury or whether injury severity predicts cardiac dysfunction. In the present study, we aimed to determine whether systolic dysfunction occurs across TBI severity in vivo through invasive hemodynamic assessment.

**FIGURE 3:**
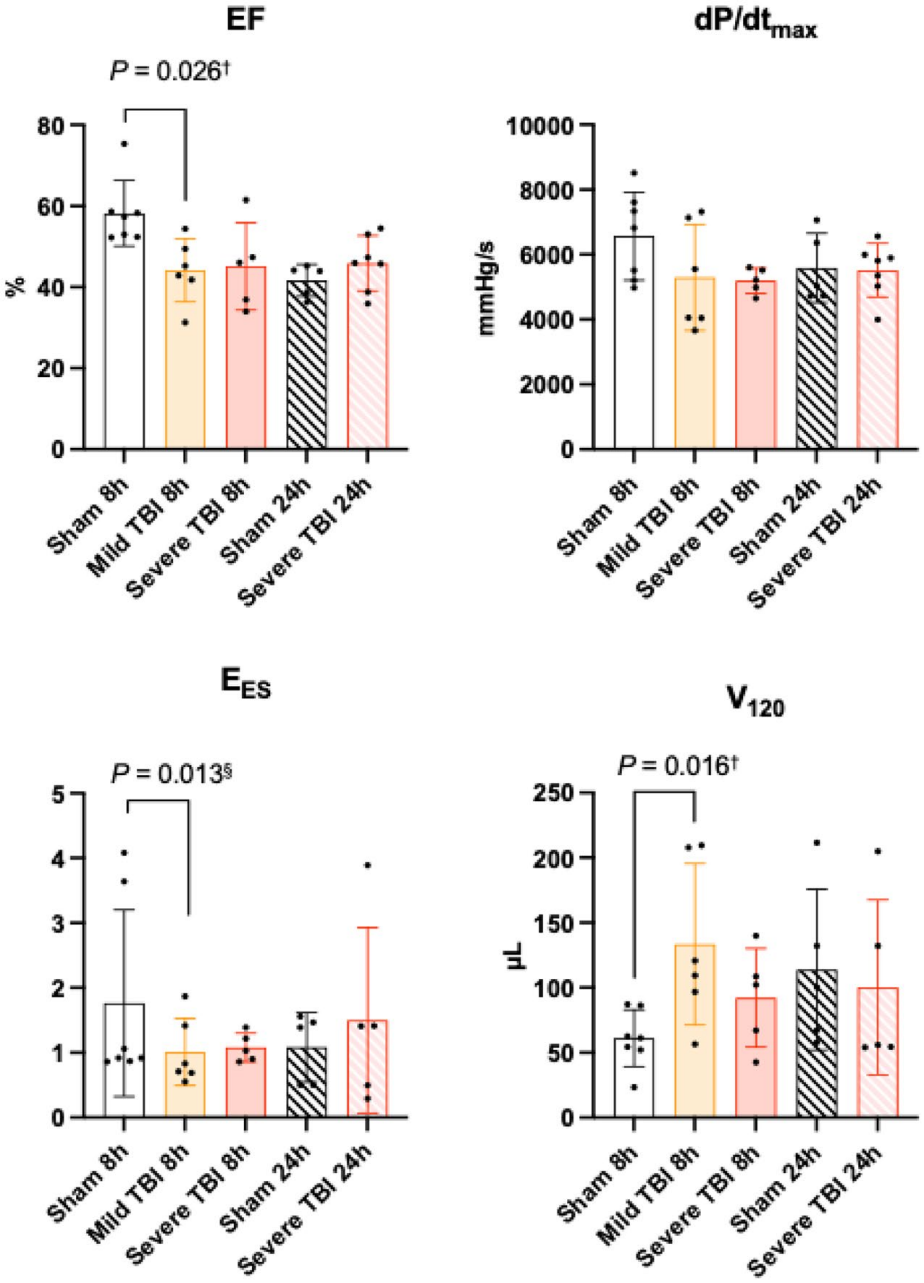
Systolic function indices. Ejection fraction (EF) and maximum rate of left ventricular pressure increase (dP/dt_max_) represent load‐dependent indices of systolic function, whereas end‐systolic elastance (E_ES_) and end‐systolic volume at 120 mmHg (V_120_) are load‐independent indices. Comparisons of EF, dP/dt_max_, and V_120_ were analyzed using one‐way ANOVA with Dunnett's post hoc correction (8 h sham vs. mild TBI, sham vs. severe TBI) and two‐way ANOVA with Sidak's post hoc correction (severe TBI 8 h–sham 8 h vs. severe–sham 24 h). E_ES_ was compared using multiple linear regression incorporating experimental group, V_0_ and (group × V_0_) as interaction terms. Displayed *p* values denote statistically significant comparisons. ^†^One‐way ANOVA with Dunnett's post hoc correction (8‐h sham vs. the indicated TBI group). ^§^Multiple linear regression incorporating experimental group, V_0_, and the group × V_0_ interaction.

We observed changes in systolic function following both mild and severe TBI; however, only mild TBI demonstrated changes in load‐independent systolic indices (Figure 3). Mild TBI was associated with decreased EF, reduced E_ES_, and increased V_120_ with a rightward shift of the PV loop. In contrast, the severe group at 8 h demonstrated trends toward reduced load‐dependent indices, including dP/dt_max_ and EF, but neither reached significance and load‐independent systolic indices (E_ES_ and V_120_) remained unchanged. Together, these findings suggest that mild and severe TBI may exhibit distinct hemodynamic responses, with alterations in load‐independent systolic indices observed following mild TBI and predominantly load‐dependent systolic changes following severe injury.

Several mechanisms may explain these severity‐dependent differences. Changes in load‐independent systolic indices after mild TBI are suggestive of altered myocardial contractile performance; however, this interpretation should be made cautiously because histologic assessment was not performed. In contrast, load‐dependent indices after severe TBI, including EF and dP/dt_max_, may reflect reduced preload and diminished Frank‐Starling recruitment at lower filling volumes (Holubarsch et al., 1996; Pérez et al., 1999).

Although E_ES_ serves as an important load‐independent index of systolic function, it has important limitations when used alone. Histologic assessment would strengthen interpretation of alterations in load‐independent systolic indices by correlating functional changes with microscopic changes in myocytes but was not performed in this study. Additionally, interpretation of E_ES_ must consider the influence of afterload and ventricular stiffness. Although relatively load‐independent, large changes in afterload may influence E_ES_ (Crottogini et al., 1994). Finally, increased LV stiffness can be associated with compensatory increases in inotropy, thus overestimating intrinsic contractility (Borlaug et al., 2009). The severe TBI group demonstrated nonsignificant trends toward increased arterial elastance and reduced ventricular compliance that could potentially influence E_ES_ despite its relative load independence. Although IVC reduction was limited to minimize reflexive sympathetic activation and preload‐related artifacts, the influence of afterload and ventricular compliance on E_ES_ cannot be excluded.

Comparison of severe TBI at 8 h and 24 h demonstrated normalization of EF at 24 h, suggesting recovery of load‐dependent systolic function. Clinical reports similarly describe rapid recovery of EF, supporting dynamic shifts in preload as a possible driver of the observed physiology (Andò et al., 2010). Despite improvement in EF, global cardiac dysfunction persisted at 24 h, suggesting that improved systolic function did not equate to complete hemodynamic recovery after severe TBI. Because mild TBI was not evaluated at 24 h, conclusions regarding the temporal differences between injury severities should be interpreted cautiously.

### 4.2 Diastolic dysfunction

Diastolic dysfunction reflects both ventricular relaxation and filling, and the latter is strongly influenced by volume status, which may be altered by TBI physiology. Hypovolemia is a common consequence after TBI, whereas diastolic dysfunction has been described with conflicting evidence (Chaikittisilpa et al., 2024; Praveen et al., 2021; Stewart et al., 2007). Additionally, hypovolemia may mimic diastolic dysfunction on echocardiography, underscoring the need for precise assessment of diastolic function after TBI.

**FIGURE 4:**
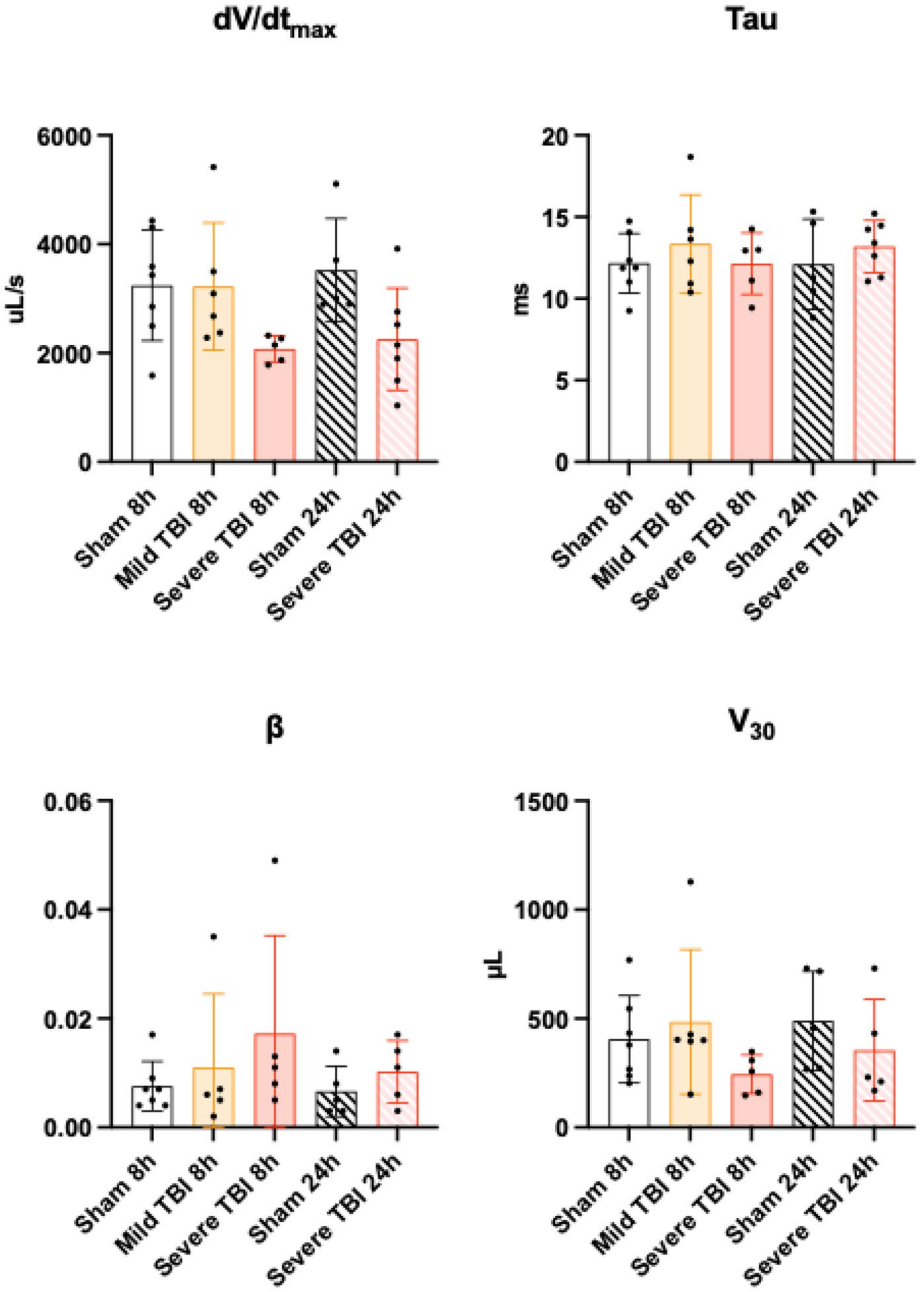
Diastolic function indices. Maximum rate of left ventricular volume increase (dV/dt_max_) and time constant of relaxation (τ) represent load‐dependent indices of diastolic function, whereas the stiffness coefficient (β) and end‐diastolic volume at 30 mmHg (V_30_ ) represent load‐independent indices. Comparisons were analyzed using one‐way ANOVA with Dunnett's post hoc correction (8 h sham vs. mild TBI, sham vs. severe TBI) and two‐way ANOVA with Sidak's post hoc correction (severe TBI 8 h–sham 8 h vs. severe–sham 24 h).

We observed abnormal diastolic function indices in the severe group at both 8 and 24 h but not in mild TBI at 8 h (Figure 4). Severe TBI was associated with decreased dV/dt_max_ and narrowing of the PV loop, but similar τ and dP/dt_min_. These findings are consistent with impaired ventricular filling and may reflect volume depletion, altered ventricular compliance, or a combination of both, with preserved isovolumetric relaxation. Hypovolemia related to cerebral salt wasting affects a substantial proportion of patients with TBI and may explain the abnormal diastolic indices observed. Volume depletion has been linked to reduced filling velocities on echocardiogram (Sugimoto et al., 2019; Tran et al., 2025), which could explain the findings observed here rather than overt diastolic dysfunction. Further, a similar volume‐restrictive pattern was seen at 24 h with normal compliance and relaxation indices, supporting hypovolemia as a plausible contributor. However, load‐independent indices of compliance (e.g., V_30_) trended worse and P_ed_ was elevated despite lower V_ed_, therefore, impaired diastolic compliance cannot be definitively excluded. Together, our findings suggest reduced LV filling likely contributes to the hemodynamic disturbances observed after severe TBI.

### 4.3 Global cardiovascular performance

Integrated measures of global cardiovascular performance reflect the overall hemodynamic consequences of neurotrauma. In contrast to assessments of systolic and diastolic function alone, global measures integrate LV performance and vascular loading conditions across multiple cardiac cycles and provide insight on overall brain–heart axis homeostasis.

**FIGURE 5:**
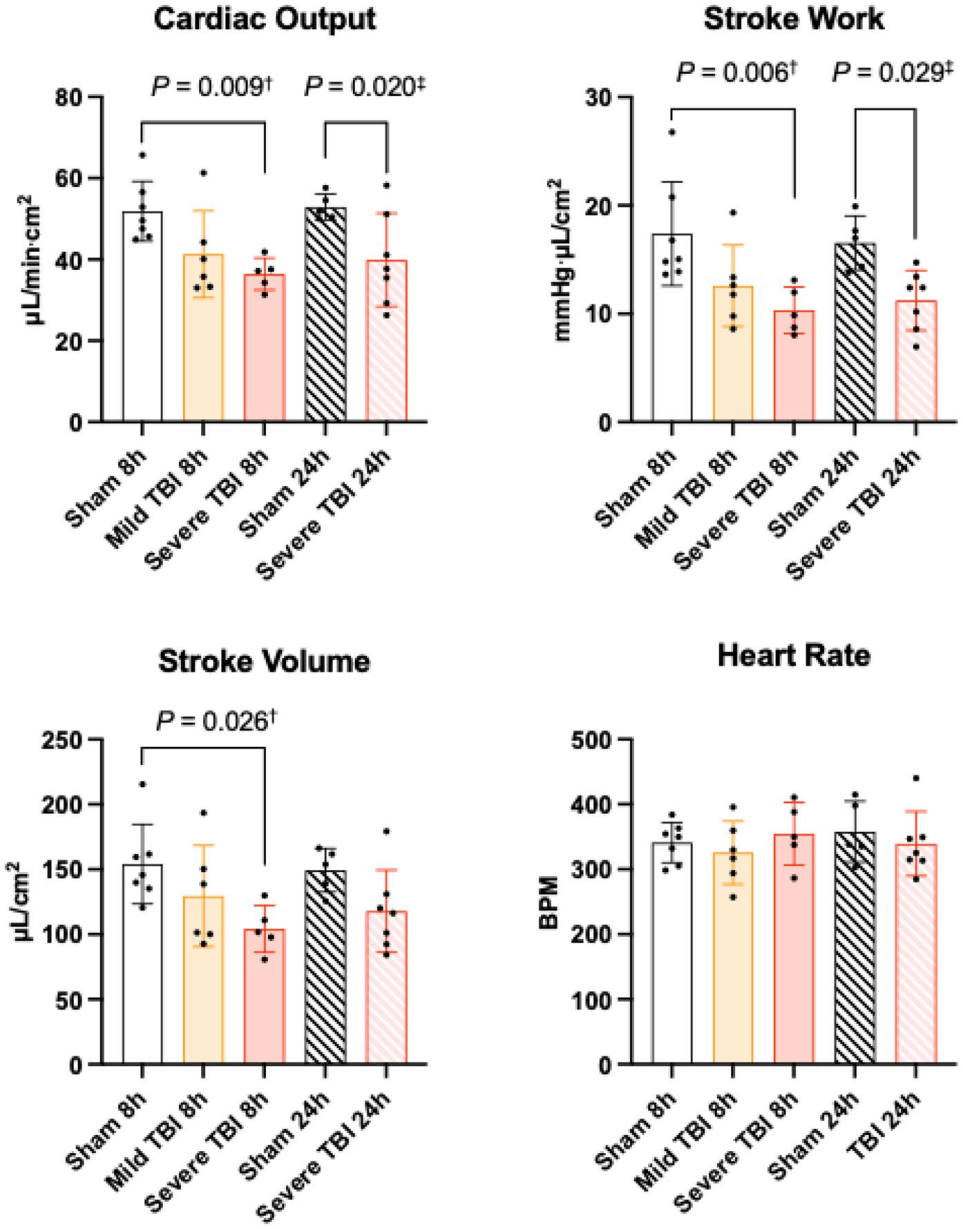
Global cardiac performance indices. Cardiac output (CO), stroke work (SW), stroke volume (SV), and heart rate (HR) represent integrated measures of global cardiovascular function. Volume‐derived measurements were indexed to rodent body surface area. Comparisons were analyzed using one‐way ANOVA with Dunnett's post hoc correction and two‐way ANOVA with Sidak's post hoc correction as described previously. Displayed *p* values denote statistically significant comparisons. ^†^One‐way ANOVA with Dunnett's post hoc correction (8‐h sham vs. the indicated TBI group). ^‡^Two‐way ANOVA with Šídák's multiple‐comparisons test (adjusted pairwise comparisons of sham vs. severe TBI at the indicated time point).

Global cardiac performance was impaired across injury severities (Figure 5), but the predominant hemodynamic pattern differed by both severity and timepoint. At 8 h, systolic dysfunction contributed to global cardiac impairment in both mild and severe TBI, with impaired LV filling further accentuating this effect in severe TBI. In contrast, severe TBI at 24 h demonstrated impaired filling with preserved systolic function. Despite reduced CO across TBI groups, HR remained similar, suggesting a potentially blunted chronotropic response to reduced cardiac output.

Neurocardiogenic reflexes are important for maintaining systemic and cerebral perfusion; however these mechanisms may be altered after TBI. Under normal conditions decreased CO stimulates a baroreceptor‐mediated sympathetic response, increasing HR and contractility to maintain organ perfusion (Benarroch, 2008). The absence of a compensatory increase in HR despite reduced CO is consistent with altered autonomic cardiovascular regulation, although autonomic function was not directly assessed in this study. Although the Cushing reflex demonstrates similar hemodynamic features in response to elevated intracranial pressure (ICP) (Fodstad et al., 2006), rodents received craniectomies that likely reduced ICP elevations associated with the injury. Therefore, the contribution of the Cushing reflex to the observed hemodynamic changes is uncertain. Because cerebral hemodynamics and autonomic function were not directly evaluated, these mechanisms warrant further investigation.

### 4.4 Echocardiography

Left ventricular wall motion is commonly assessed by echocardiography, and regional wall motion abnormalities and hypokinesia have been reported after TBI. The regional distribution may be informative, as Takotsubo's cardiomyopathy (TC) is typically described with characteristic apical hypokinesia (apical ballooning), whereas basal or global involvement has been described in neurogenic stunned myocardium (NSM) (Ancona et al., 2016; Ohtsuka et al., 2000; Singh et al., 2022). Because TC and NSM share similar hypothesized mechanisms, they are often considered along a spectrum of stress cardiomyopathies, and distinguishing between them can be challenging.

We observed global hypokinesis more frequently in the mild TBI group at 8 h than the severe TBI group. We observed apical hypokinesia in one rodent in both severe TBI groups (8 h and 24 h), and also one rodent in the sham group at 24 h. Although the predominantly global hypokinesis pattern may be more consistent with patterns described in NSM than TC, these qualitative findings should be interpreted with caution. Echocardiographic data were not interpreted in a blinded manner and therefore should not be considered definitive evidence of regional or global myocardial dysfunction. Rather, the invasive hemodynamic data provide the primary evidence of cardiac dysfunction, while these exploratory echocardiographic observations may inform future studies, incorporating blinded image analysis and more rigorous assessment of regional wall motion to better characterize the phenotype of TBI‐associated cardiac dysfunction.

### 4.5 Serum catecholamines

Catecholamine‐mediated cardiac dysfunction is commonly linked to neurogenic cardiomyopathies, including TC and NSM. Clinical evidence supporting this mechanism is most clearly described in TC, in which severe emotional or physical stress triggers autonomic hyperactivation and substantial elevations in circulating catecholamines (Wittstein et al., 2005). In contrast, cardiac dysfunction after TBI has been reported without substantial elevations in serum catecholamines (Banki et al., 2005; Tung et al., 2004), suggesting the relationship between circulating catecholamines and TBI‐associated cardiac dysfunction is unclear.

**FIGURE 6:**
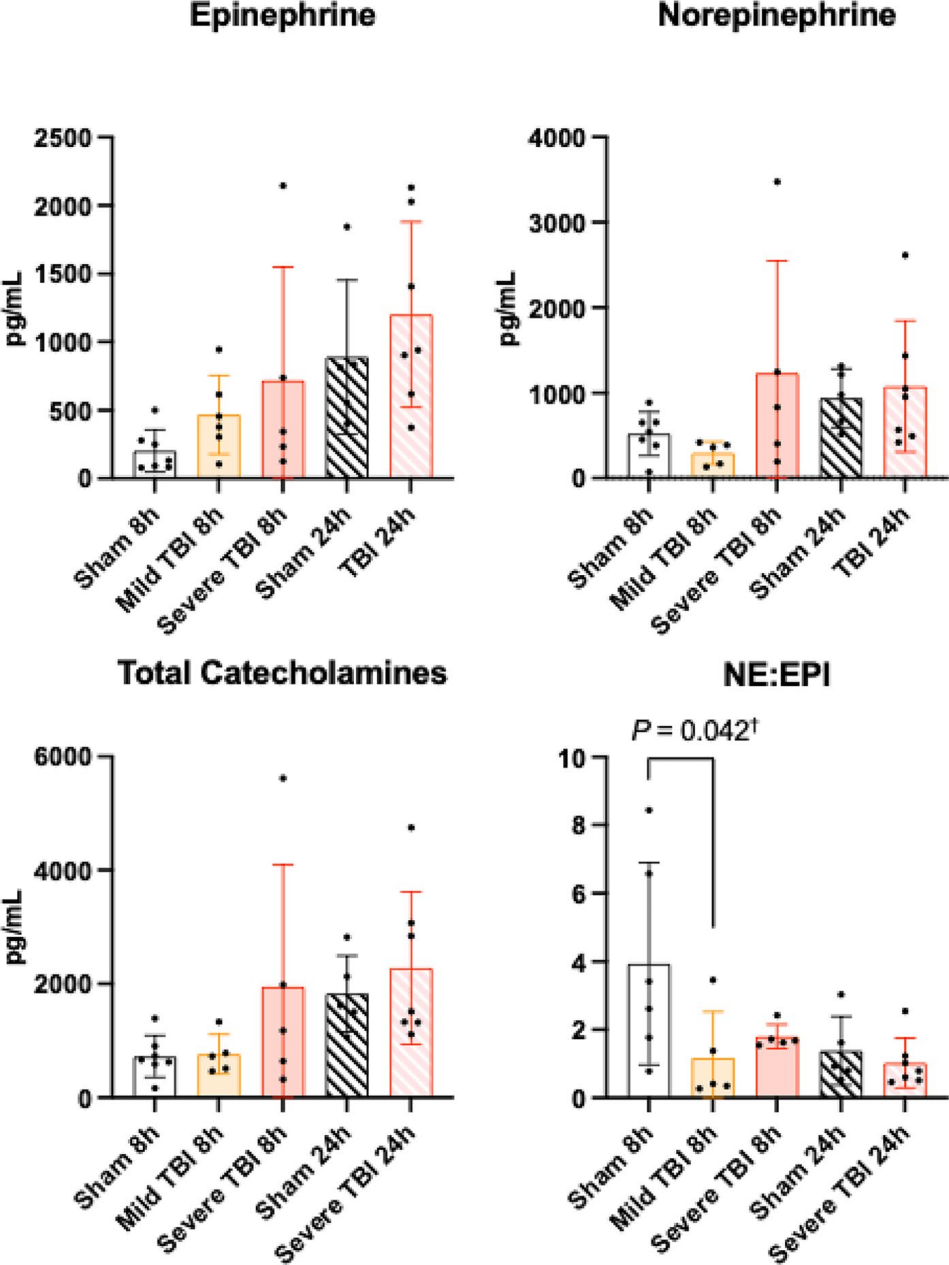
Serum catecholamine profiles. Serum epinephrine (EPI), norepinephrine (NE), total catecholamines (EPI + NE), and the NE‐to‐EPI ratio (NE:EPI) were quantified at 8 and 24 h after TBI. Comparisons were analyzed using one‐way ANOVA with Dunnett's post hoc correction and two‐way ANOVA with Sidak's post hoc correction as described previously. Displayed *p* values denote statistically significant comparisons. ^†^One‐way ANOVA with Dunnett's post hoc correction (8‐h sham vs. the indicated TBI group).

In our study, serum catecholamines did not differ between TBI and shams; however the NE:EPI was lower in mild TBI after 8 h (Figure 6). These findings suggest that circulating catecholamines alone may not fully account for the observed cardiovascular alterations, although the potential contribution of catecholaminergic balance warrants further investigation (Redfors et al., 2014). Despite correlating hemodynamic indices with circulating catecholamines, we did not correlate serum catecholamine concentrations with histology; therefore, we cannot draw conclusions regarding the underlying myocardial structural changes or other mechanistic inferences.

### 4.6 Alternative mechanisms of cardiac dysfunction

In addition to the role of circulating catecholamines, other systemic and neurologic mechanisms may contribute to cardiac dysfunction after TBI. Direct stimulation of the myocardium by postganglionic sympathetic fibers may influence cardiac function in ways not captured by measurements of serum catecholamines alone (Brodde & Michel, 1999). Furthermore, the inflammatory response associated with TBI may disrupt microvascular function and alter myocardial function. Inflammatory‐mediated endothelial dysfunction and impaired nitric oxide synthesis may alter vascular tone and influence afterload (Villalba et al., 2017). Experimental models have also demonstrated increased inflammatory cytokines and inflammasome activation within cardiomyocytes, suggesting potential immune‐mediated mechanisms that contribute to cardiac dysfunction (Keane et al., 2023). Neuroglial release of exosomes after TBI has also been proposed to target the heart and has been associated with cardiomyocyte injury and cell death (Ko et al., 2020; Xiao et al., 2019; Yao et al., 2020). Although these mechanisms remain speculative in the context of the present study, together they suggest that neurogenic and inflammatory pathways may contribute to cardiac dysfunction after TBI and highlight the need for further studies integrating mechanistic and hemodynamic assessments.

### 4.7 Limitations

We acknowledge several limitations to our study design. First, our sample size was modest, and no a priori power calculations were performed. Although we detected significant differences in several hemodynamic parameters, the study may have been underpowered to detect more subtle differences in secondary outcomes, including diastolic indices and serum catecholamine measurements. Accordingly, nonsignificant findings should be interpreted cautiously as type II error cannot be excluded. Second, data analysis and echocardiographic interpretation were not performed in a blinded fashion, which introduces the potential for observer bias. Although PV‐loop analysis provides objective computational data output, echocardiographic assessment may be subjective and operator dependent, particularly when evaluating wall‐motion abnormalities. Third, although we included analysis of severe TBI cohorts at both 8 and 24 h, mild TBI was not evaluated at 24 h, limiting temporal characterization across both injury severities. Additionally, E_ES_ was analyzed using separate multiple linear regression models for the 8 h and 24 h cohorts rather than a combined model including time, because inclusion of time introduced substantial multicollinearity (high variance inflation factor), limiting our ability to directly model temporal effects. Fourth, although we present detailed hemodynamic analysis including load‐independent indices, we did not directly measure volume status, intracranial pressure, cerebral hemodynamics, or autonomic function. As a result, we cannot definitively determine the relative contributions of altered myocardial contractility, neurogenic reflexes, or dynamic loading conditions to the observed cardiovascular changes. Finally, histologic analyses were not performed. Therefore, we cannot directly correlate hemodynamic findings with contraction band necrosis, mitochondrial injury, or inflammatory changes, limiting mechanistic interpretation of the observed cardiovascular alterations.

## 5 CONCLUSION

TBI was associated with cardiovascular dysfunction that varied according to injury severity. Using a CCI model combined with invasive PV‐loop analysis, we identified changes in load‐independent systolic indices following mild TBI and persistent impairment of global cardiac performance following severe TBI. Although this study characterized the time course of severe TBI, future studies are needed to determine how cardiac dysfunction evolves following mild TBI. Qualitative echocardiographic findings complemented the invasive hemodynamic assessment, whereas circulating catecholamine concentrations were not significantly different from controls.

Together, these findings demonstrate the value of controlled experimental models for characterizing brain‐heart interactions after TBI and provide a foundation for future studies investigating the mechanisms underlying TBI‐associated cardiac dysfunction.

## AUTHOR CONTRIBUTIONS

**Preston So:** Data curation; formal analysis; investigation; project administration; software; validation; visualization. **Amit Iyengar:** Conceptualization; data curation; formal analysis; investigation; methodology; software; supervision; validation; visualization. **Noah Weingarten:** Conceptualization; data curation; formal analysis; investigation; methodology; software; validation. **Mrinal Patel:** Conceptualization; data curation; investigation; methodology; software. **Jessica Dominic:** Conceptualization; data curation; formal analysis; investigation; methodology; project administration; software; visualization. **Andrew Belec:** Investigation; software. **Joyce Ho:** Investigation; software. **Samuel S. Shin:** Conceptualization; funding acquisition; investigation; methodology; resources; supervision; validation. **Pavan Atluri:** Conceptualization; funding acquisition; investigation; methodology; resources; supervision.

## FUNDING INFORMATION

This work was supported by departmental funds from the University of Pennsylvania Department of Cardiovascular Surgery.

## CONFLICT OF INTEREST STATEMENT

The authors declare no conflicts of interest.

## ETHICS STATEMENT

All experiments were conducted in accordance with protocols approved by the Institutional Animal Care and Use Committee (IACUC) at the University of Pennsylvania and with the National Institutes of Health Guide for the Care and Use of Laboratory Animals. Animals were housed and acclimatized under appropriate conditions, and all efforts were made to minimize unnecessary pain and distress throughout the experimental procedures.

## Data Availability

The data supporting the findings of this study are available from the corresponding author upon reasonable request.
